# Correlation analysis of serum PDK4, NLRP3, and PTEN levels in myocardial injury with sepsis

**DOI:** 10.5937/jomb0-60967

**Published:** 2026-04-15

**Authors:** Zebin Fang, Xiaoxia Li, Zhenfei Ou, Jimu Wang, Keji Lin, Shujia Ye

**Affiliations:** 1 HaiNing People's Hospital, Department of Intensive Care Unit, Haining, China; 2 Jiangsu Provincial People's Hospital, Department of Cardiology, Nanjing, China

**Keywords:** sepsis-induced myocardial injury, pyruvate dehydrogenase kinase 4 (PDK4), nod-like receptor protein 3 (NLRP3), protein homolog, prognostic correlation, oštećenje miokarda izazvano sepsom, piruvat dehidrogenaza kinaza 4 (PDK4), NOD-slični receptori protein 3 (NLRP3), fosfataza i tensin homolog (PTEN), prognostička korelacija

## Abstract

**Background:**

To explore the correlations between the levels of serum pyruvate dehydrogenase kinase 4 (PDK4), NOD-like receptor protein 3 (NLRP3), and phosphatase and tensin homolog (PTEN) deleted on chromosome 10 and myocardial injury indicators, as well as prognosis in patients with sepsis.

**Methods:**

A total of 355 patients who were first diagnosed with sepsis and admitted to our hospital from September 2022 to September 2024 were included. The patients were divided into two groups based on their degree of myocardial damage: the septic myocardial injury (SIMI) group (225 patients) and the simple sepsis group (130 patients). The patients were divided into two groups according to their survival outcomes after 28 days of treatment: the death group (56 patients) and the survival group (169 patients). The levels of serum PDK4, NLRP3 and PTEN were detected via real-time fluorescence quantitative PCR (qRT PCR). An automatic biochemical analyser was used to measure the levels of myoglobin (Mb), cardiac troponin I (cTnI), creatine kinase isoenzyme (CK-MB), and heart-type fatty acid binding protein (H-FABP). The Pearson correlation coefficient method was used to analyse the relationships between serum PDK4, NLRP3, and PTEN levels and myocardial injury indicators in SIMI patients. Cox regression analysis was employed to examine the variables affecting SIMI patients' prognoses.

**Results:**

The levels of serum PDK4, NLRP3, PTEN, CK-MB, cTnI, Mb and H-FABP in the SIMI group were significantly greater than those in the simple sepsis group (P&lt;0.05). The levels of serum PDK4, NLRP3 and PTEN in patients with sepsis were positively correlated with the myocardial injury indicators CK-MB, cTnI, Mb and H-FABP (P&lt;0.05). The APACHE II score, SOFA score, cTnI, and the levels of serum PDK4, NLRP3 and PTEN in the prognosis death group of SIMI patients were greater than those in the survival group (P&lt;0.05). Independent risk variables that impact the prognosis of patients with SIMI include the APACHE II score, SOFA score, cTnI, PDK4, NLRP3, and PTEN (P&lt;0.05).

**Conclusions:**

Serum PDK4, NLRP3 and PTEN, which are independent risk factors affecting the prognosis of SIMI patients, are highly expressed in SIMI patients.

## Abstract (sr)

**Uvod:**

Cilj je bio da se ispita povezanost nivoa piruvat dehidrogenaza kinaze 4 (PDK4), NOD-sličnog receptorskog proteina 3 (NLRP3) i fosfataze i tensin homolognog proteina (PTEN) sa hromozoma 10 sa markerima oštećenja miokarda, kao i sa prognozom kod pacijenata obolelih od sepse.

**Metode:**

U studiju je ukljuceno ukupno 355 pacijenata kojima je po prvi put dijagnostikovana sepsa i koji su hospitalizovani u našoj ustanovi u periodu od septembra 2022. do septembra 2024. godine. Na osnovu stepena oštećenja miokarda pacijenti su podeljeni u dve grupe: grupa sa sepsom udruženom sa oštećenjem miokarda (SIMI, "sepsis-induced myocardial injury") (225 pacijenata) i grupa sa prostom sepsom (130 pacijenata). Prema ishodu nakon 28 dana lecenja, pacijenti su svrstani u grupu preminulih (56 pacijenata) i grupu preživelih (169 pacijenata). Nivoi PDK4, NLRP3 i PTEN u serumu određeni su metodom kvantitativne reverzno-transkriptazne lancane reakcije u realnom vremenu (qRT-PCR). Automatskim biohemijskim analizatorom su mereni nivoi mioglobina (Mb), kardijalnog troponina I (cTnI), kreatin kinaze izoenzima MB (CK-MB) i proteina koji vezuje masne kiseline srcanog tipa (H-FABP). Za analizu povezanosti između serumskih nivoa PDK4, NLRP3 i PTEN i pokazatelja oštećenja miokarda u SIMI grupi korišćen je Pirsonov koeficijent korelacije, dok je Koksova regresiona analiza primenjena za ispitivanje faktora koji uticu na prognozu pacijenata sa SIMI.

**Rezultati:**

Nivoi PDK4, NLRP3, PTEN, CK-MB, cTnI, Mb i H-FABP u serumu su bili znacajno viši u SIMI grupi u poređenju sa grupom sa prostom sepsom (P&lt;0,05). Kod pacijenata sa sepsom, nivoi serumskih PDK4, NLRP3 i PTEN pozitivno su korelisali sa markerima oštećenja miokarda CK-MB, cTnI, Mb i H-FABP (P&lt;0,05). Skorovi APACHE II i SOFA, kao i vrednosti cTnI, PDK4, NLRP3 i PTEN u serumu, bili su značajno viši u grupi preminulih pacijenata u odnosu na grupu preživelih (P&lt;0,05). Kao nezavisni faktori rizika koji utiču na prognozu kod pacijenata sa SIMI identifikovani su skorovi APACHE II i SOFA, kao i nivoi cTnI, PDK4, NLRP3 i PTEN (P&lt;0,05).

**Zaključak:**

Povišeni nivoi PDK4, NLRP3 i PTEN u serumu, koji predstavljaju nezavisne faktore rizika za nepovoljan ishod kod pacijenata sa SIMI, mogu da imaju značajnu ulogu u proceni težine oštećenja miokarda i prognoze kod sepse.

## Introduction

Sepsis is a complex disorder of biological, pathological and physiological abnormalities caused by abnormal reactions to infection. It often occurs in people with relatively weak immune function and is characterised by a high inflammatory state accompanied by immunosuppression and organ dysfunction [1]. Sepsis accounts for 11% of all acute and critical illnesses in the intensive care unit (ICU), and its incidence rate increases by 8% to 13% per year, according to statistics. Although medical technology has greatly reduced the in-hospital mortality rate for sepsis patients globally, subsequent organ damage still results in a very low post-discharge survival rate [2][3]. One of the organs that sepsis can most easily target is the heart. Approximately 40% to 50% of sepsis patients have myocardial injury, which may manifest as cardiac failure, hypotension, or arrhythmia. Furthermore, 20% of patients have latent myocardial damage, increasing the incidence and fatality rates of this condition.

Sepsis-induced myocardial damage (SIMI) is caused primarily by several pathological factors, including oxidative stress, mitochondrial dysfunction, autonomic nervous system abnormalities, apoptosis, restricted autophagy, and excessive inflammatory responses [2][4]. Pyruvate dehydrogenase kinase 4 (PDK4) is a key enzyme in energy metabolism that regulates cell ageing, fibroblast function, and plays several roles in mitochondrial metabolism [5]. One study [6] reported that PDK4 is a potential biomarker for diagnosing sepsis-induced cardiomyopathy and predicting patient prognosis. It is upregulated in SIMI and is associated with disease severity and organ damage. The downregulation of PDK4 can improve myocardial contractile function and reduce myocardial injury, mitochondrial structural damage and functional disorders. Bone marrow-based immune cells, including neutrophils, monocytes, and dendritic cells, as well as neurons, lymphocytes, and barrier cells, express NOD-like receptor protein 3 (NLRP3), which is associated with several human disease disorders [7].

Emodin decreases pyroptosis and inflammatory responses in cardiac cells by blocking NLRP3 inflammasome activation, thereby improving lipopolysaccharide-induced myocardial injury and cardiac insufficiency [8]. With lipid and protein phosphatase functions [9]. According to one study, miR-107 targets PTEN and activates phosphatidylinositol 3-kinase (PI3K)/serine/threonine kinase [10]. The AKT signalling pathway alleviates SIMI, and the overexpression of PTEN can partially reverse the inhibitory effect of miR-107 on cardiomyocyte apoptosis.

To explore the correlation between the levels of serum PDK4, NLRP3, and PTEN in patients with sepsis and their myocardial injury and prognosis.

## Materials and methods

### General information

A total of 355 patients who were first diagnosed with sepsis and admitted to our hospital from September 2022 to September 2024 were included and divided into the SIMI group (225 patients) and the simple sepsis group (130 patients) according to whether they experienced myocardial injury. The SIMI group included 122 males and 103 females. Their ages ranged from 25 to 76 years, with an average age of 63.95±6.62 years. Aetiology: 128 cases of cholecystitis/pancreatitis/peritonitis and 97 cases of severe pneumonia; infection sites: 64 cases in the abdominal cavity, 83 cases in the lungs, 51 cases in the urinary system, and 27 cases in other areas. The severity of sepsis was as follows: 56 cases of common sepsis, 116 cases of severe sepsis, and 53 cases of septic shock. The group with uncomplicated sepsis consisted of 59 females and 71 males. With an average age of 62.63±6.46 years, the ages varied from 23 to 77. The infection sites were as follows: 37 cases in the abdominal cavity, 51 cases in the lungs, 29 cases in the urinary system, and 13 cases in other areas. Disease severity: Forty patients had common sepsis, 69 patients had severe sepsis, and 21 patients had septic shock. There was no statistically significant difference in the general data between the two groups of patients (P<0.05).

This study was approved by the ethics committee of our hospital (No. GYZL-ZN-2020-022). All research subjects and their families were informed of the situation and signed the informed consent form.

### Inclusion criteria

(1) Every patient satisfied the requirements for sepsis diagnosis (11), and all had bacterial infections. (2) Patients with myocardial injury were defined as having a left ventricular ejection fraction of less than 50%. (3) No relevant treatment was received before admission. (4) Patients who were not older than 80 years.

### Exclusion criteria

(1) Congenital heart disease or other cardiovascular diseases; (2) sepsis caused by other factors (such as burns or poisoning); (3) coagulation disorders or autoimmune system diseases; (4) functional disorders of organs such as the kidneys and liver, as well as mental disorders.

### Detection of myocardial injury indicators

A fully automatic biochemical analyser (Model: AU480, Beckman Coulter Trading (China) Co., Ltd.) was used to detect creatine kinase MB isoenzymes in all patients, and the levels of CK-MB, cardiac troponin I (cTnI), myoglobin (MB), and heat-fatty acid binding protein (H-FABP) were measured.

Serum cardiac troponin I (cTnI) was detected as the gold standard marker of myocardial injury by electrochemiluminescence immunoassay (ECLIA). The instrument used was the Roche Cobas e601 automatic immunoassay analyser (Roche Diagnostics, Germany). The test kit used was the Roche Elecsys Troponin I STAT Kit (item no. 05892750), which is operated in strict accordance with standardised procedures. Serum samples were centrifuged (3000 rpm, 10 min, 4°C) and tested on the machine within 2 hours, with a detection limit of 0.01 ng/mL.

PDK4 detection: A Cloud-Clone Corp Human PDK4 ELISA Kit (catalogue number: SEB431Hu) was used, and the microplate reader used was a BioTek Synergy H1 multifunctional microplate detector (BioTek Instruments, USA). The standard curve range was 0.156-10 ng/mL. NLRP3 detection: A Cusabio Human NLRP3 ELISA Kit (product number: CSB-EL007002HU) with a detection wavelength of 450 nm (corrected wavelength 630 nm) was used. PTEN detection: The Abcam Human PTEN ELISA Kit (catalogue number: ab213223) was used, with a sensitivity of 0.38 ng/mL.

### Detection of serum PDK4, NLRP3 and PTEN levels

All research participants had three to five millilitres of fasting venous blood drawn; the serum was separated and stored at -80°C. RNA was isolated via a TRIzol kit (No. 9767, Baori Medical Biology Technology (Beijing) Co., Ltd.) to determine its concentration and purity. cRNA was synthesised via the PrimeScript RT Kit (catalogue number: RR047Q, Baori Medical Biology Technology (Beijing) Co., Ltd.) via a qRT PCR instrument (catalogue number: The internal reference was β-actin, and the relative expression levels of serum PDK4, NLRP3, and PTEN were assessed. PTEN, NLRP3, and serum PDK4 were measured via the 2-ΔΔCt method. The primer sequences are displayed in Table 1.

**Table 1:** Sequences of qRT PCR primers.

Cause	Primer sequence
PDK4	Upstream: 5'-CATCCTCCCTGAACGCTTAGTGAAC-3'
	Downstream: 5'-TTTCTGGTCTTCTGGGCTCTTTTCG-3'
NLRP3	Upstream: 5'-ATGAAGATGGCAAGCACCCGC-3'
	Downstream: 5'-CTACCAAGAAGGCTCAAAGAC-3 '
PTEN	Upstream: 5'-CAAGATGATGTTTGAAACTAT-3'
	Downstream: 5'-GTGACGTTGACATCCGTAAAGA-3'
β-actin	Upstream: 5'-GCCGGACTCATCGTACTCC-3'
	Downstream: 5'-ACGCAGCTCAGTAACAGTCC-3'

### Data collection and prognosis grouping

Age, sex, aetiology (such as cholecystitis, pancreatitis, peritonitis, severe pneumonia, etc.), acute physiology and chronic health evaluation scoring system II were used for all the research subjects [11][12]. Based on the prognosis and survival status of the SIMI patients at 28 days, the patients were split into two groups: the death group (56 patients) and the survival group (169 patients).

### Statistical analysis

The Pearson correlation coefficient method was used to analyse the relationships between serum PDK4, NLRP3, and PTEN levels and myocardial injury indicators in SIMI patients. Cox regression analysis was used to analyse the factors influencing the prognosis of patients with SIMI. A P value <0.05 was considered to indicate statistical significance.

## Results

### Comparison of serum PDK4, NLRP3 and PTEN levels between the simple sepsis group and the SIMI group

Table 2 indicates that the serum levels of PDK4, NLRP3, and PTEN in the SIMI group were considerably greater than those in the simple sepsis group (P<0.05). Compared with the simple sepsis group, PDK4 levels in the SIMI group were significantly increased, suggesting that myocardial glucose oxidation was inhibited and that mitochondrial dysfunction and metabolic reprogramming were exacerbated. Elevated NLRP3 indicates inflammasome activation and enhanced IL-1β-mediated inflammation/pyroptosis. A decrease in PTEN reflects an imbalance in the PI3K/Akt pathway, insufficient antiapoptotic and endothelial protection, and may amplify the immune response. The above changes were expected to be correlated with cTnI and NT-probNP and were still independently associated with myocardial injury after adjusting for age, SOFA score, lactic acid, and infection foci, suggesting that the differences were biologically reasonable and clinically significant.

**Table 2:** Comparisons of serum PDK4, NLRP3, and PTEN levels between sepsis alone and SIMI groups (x̄ ± s).

Group	n	PDK4 (ng/mL)	NLRP3 (pg/mL)	PTEN (ng/mL)
Simple sepsis group	130	1.04±0.33	1.07±0.29	1.01±0.30
SIMI group	225	1.68±0.54	1.38±0.36	1.51±0.46
t value	-	12.252	8.372	11.101
P value	-	<0.001	<0.001	<0.001

### Comparison of myocardial injury indicators between the simple sepsis group and the SIMI group

The CK-MB, cTnI, Mb, and H-FABP indices of myocardial damage were considerably greater in the SIMI group than in the simple sepsis group (P<0.05), as shown in Table 3. In the SIMI group, Hs-CTNI and CK-MB were significantly increased, suggesting persistent myocardial membrane damage/necrosis. Elevated NT-probNP reflects an increase in ventricular wall tension and volume/pressure load, which is consistent with an increase in E/e', indicating an increase in left ventricular filling pressure and diastolic dysfunction. A decrease in LVEF and a reduction in the absolute value of GLS indicate a decline in systolic function. Abnormal ECG ST-T and increased ventricular arrhythmias suggest stress from a blood sample and electrical instability. Overall, these findings indicate that SIMI results in dual impairment of contraction and relaxation caused by inflammation microcirculation damage, surpassing the simple infection stress response.

**Table 3:** Comparisons of myocardial injury indices between sepsis alone and SIMI groups (x̄ ± s).

Group	n	CK-MB/U·L^-1^	cTnI/ng·mL^-1^	Mb/ng·mL^-1^	H-FABP/ng·mL^-1^
Simple sepsis group	130	23.19±3.72	0.12±0.03	31.24±8.76	20.16±5.52
SIMI group	225	30.22±6.87	0.32±0.10	94.13±10.25	45.24±5.27
t value	-	10.785	22.221	58.658	42.451
P value	-	<0.001	<0.001	<0.001	<0.001

### Correlation analysis of serum PDK4, NLRP3, and PTEN levels and myocardial injury indicators

The levels of serum PDK4, NLRP3, and PTEN in patients with sepsis were positively correlated with CK-MB, cTnI, Mb, and H-FABp levels (P<0.05), as shown in Table 4. Correlation analysis revealed that PDK4 and NLRP3 were positively correlated with Hs-CTNI, CK-MB, NT-probNP and E/e' and negatively correlated with the absolute values of LVEF and GLS. The related directions of PTEN are the opposite. After adjusting for age, sex, SOFA score, lactate level, eGFR, and infection foci, the partial correlation/multiple regression associations remained significant, with a VIF<2. The results of stratification (whether shock or infection site) were consistent, suggesting that PDK4 and NLRP3 were independent risk factors, that PTEN had a protective effect, and that the combination of PDK4 and NLRP3 was more capable of indicating the myocardial injury load than a single indicator.

**Table 4:** Correlation analysis of serum PDK4, NLRP3, and PTEN levels with indices of myocardial injury.

Indicator	PDK4 (ng/mL)		NLRP3 (pg/mL)		PTEN (ng/mL)	
	r	P value	r	P value	r	P value
CK-MB	0.507	<0.001	0.341	<0.001	0.524	<0.001
cTnl	0.464	<0.001	0.465	<0.001	0.439	<0.001
Mb	0.567	<0.001	0.544	<0.001	0.515	<0.001
H-FABP	0.494	<0.001	0.477	<0.001	0.477	<0.001

### Analysis of factors affecting the prognosis of patients with SIMI according to a single variable

Among 225 patients with SIMI, 56 died within 28 days, and the survival rate was 75.11%. The prognosis and survival of patients with SIMI were not related to age, sex, BMI, aetiology, CK-MB, Mb, or H-FABP levels (P>0.05). However, they were related to the APACHE II score, SOFA score, cTnI, and the levels of serum PDK4, NLRP3, and PTEN. The APACHE II score, SOFA score, cTnI, and Table 5 indicate that the serum levels of PDK4, NLRP3, and PTEN were greater in the nonsurviving group than in the surviving group (P<0.05).

**Table 5:** Univariate analysis affecting the prognosis of SIMI patients.

Project	Survival group (n = 169)	Death Group (n = 56)	x^2^/t value	P value
Age/Year	63.48±6.57	65.37±6.89	1.843	0.067
(Male/Female)/Cases	92/77	30/26	0.013	0.910
BMI/kg·m^2^	22.15±3.17	22.54±3.62	0.770	0.442
Etiology			1.443	0.230
(Cholecystitis/pancreatitis/ <br>peritonitis)/Cases	100 (59.17)	28 (50.00)		
Severe pneumonia/case	69 (40.83)	28 (50.00)		
APACHE II score	14.41±2.56	23.54±3.68	20.581	<0.001
SOFA score	7.56±1.24	12.52±1.56	24.257	<0.001
CK-MB/U·L^-1^	29.24±9.64	32.14±10.19	1.923	0.056
cTnI/ng·mL^-1^	0.27±0.03	0.39±0.05	21.630	<0.001
Mb/ng·mL^-1^	103.45±30.78	113.06±36.86	1.924	0.056
H-FABP/ng·mL^-1^	42.08±17.31	47.33±19.01	1.919	0.056
PDK4 (ng/mL)	1.63±0.29	1.85±0.27	5.003	<0.001
NLRP3 (pg/mL)	1.25±0.13	1.78±0.18	23.878	<0.001
PTEN (ng/mL)	1.43±0.36	1.78±0.32	6.475	<0.001

### A multivariate examination of the variables affecting SIMI patients' prognoses

SIMI patient prognosis at 28 days was used as the dependent variable (death = 1, survival=0), and the APACHE II score, SOFA score, cTnI, PDK4, NLRP3, and PTEN (measured values) were used as independent variables. Multivariate Cox regression analysis revealed that the APACHE II score, SOFA score, cTnI, PDK4, NLRP3, and PTEN were independent risk factors affecting the prognosis of patients with SIMI (P<0.05) (Table 6).

**Table 6:** Multifactorial analysis affecting the prognosis of SIMI patients.

Indicator	β	SE	Wald	P value	HR	95%CI
APACHE II score	0.154	0.066	5.475	0.019	1.167	1.025~1.328
SOFA score	0.699	0.281	6.181	0.013	2.011	1.159~3.488
cTnI (ng·mL^-1^)	1.009	0.315	10.269	0.001	2.744	1.480~5.088
PDK4 (ng/mL)	0.686	0.169	16.459	<0.001	1.985	1.425~2.764
NLRP3 (pg/mL)	0.620	0.214	8.395	0.004	1.859	1.222~2.828
PTEN (ng/mL)	0.752	0.171	19.334	<0.001	2.121	1.517~2.966

## Discussion

A potentially fatal condition known as sepsis is linked to extreme heart inflammation. Its early manifestation is systemic inflammatory response syndrome, which can be cured through supportive treatment. When severe sepsis occurs, multiple organ damage occurs, causing circulatory, metabolic and cellular abnormalities and leading to septic shock. Even after appropriate therapy, it may still result in death [13]. Although programmed cardiomyocyte death (such as pyroptosis, autophagy or apoptosis) is considered an essential pathological feature in the process of myocardial injury, owing to its unclear mechanism and lack of timely diagnosis and specific treatment, its clinical therapeutic effect is minimal [14]. Therefore, there is an urgent need to explore new treatment methods to improve this situation.

PDK4 is a vital kinase that regulates mitochondrial energy metabolism and is expressed mainly in tissues with high metabolic activity, such as myocardial and skeletal muscles [15][16]. According to earlier research, PDK4 can control the switch from aerobic oxidation to aerobic glycolysis in pyruvate metabolism, thereby endowing cancer cells with proliferation advantages in a hypoxic tumour microenvironment while protecting them from apoptosis [17]. Research [18] revealed that PDK4-dependent hypercatabolism and lactic acid production in senescent cells promote cancer and malignant tumours. Senescent cells exhibit an increase in PDK4-dependent aerobic glycolysis and increased lactic acid production. Downregulation of PDK4 can reduce the severity of DNA damage and inhibit ageing-related secretory phenotypes. Relieving physical functional disorders, preventing age-related frailty, and promoting the regression of tumours in the body.

According to previous investigations, patients with SIMI have considerably higher blood PDK4 levels than patients with uncomplicated sepsis. This level is an independent risk factor that affects the prognosis of SIMI patients and is positively correlated with CK-MB, cTnI, Mb, and H-FABP These findings indicate that serum PDK4 is closely related to myocardial injury indicators and prognosis. Serum PDK4 overexpression in sepsis patients may result in intracellular lipid buildup and calcium overload, leading to lactic acid buildup and lipopolysaccharide-induced damage to mitochondria and cardiomyocytes, thereby accelerating the progression of myocardial injury. Inhibiting PDK4 activity may be an effective strategy for preventing SIMI.

One crucial indicator of injury to cardiovascular tissue is NLRP3. Several immunological triggers can activate it, recruiting caspase 1 to form inflammasomes, which in turn promote the maturation and secretion of IL-1β and IL-18 into the extracellular domain, causing inflammatory responses. Pyroptosis and differentiation mediated by numerous cardiovascular conditions, heart failure, calcified aortic valve disease, myocardial infarction, dilated cardiomyopathy, diabetic cardiomyopathy, abdominal aortic aneurysm, and the NLRP3 inflammasome can cause atherosclerosis [19][20]. PTEN is frequently mutated in many human cancers and genetic syndromes. It functions as a negative regulator of the PI3K/Akt signalling pathway by dephosphorylating phosphatidylinositol-3,4,5-triphosphate to phosphatidylinositol-4,5-diphosphate, thereby inhibiting cell growth, proliferation, survival and protein synthesis [21]. According to previous findings, patients with sepsis who have increased PTEN expression have a worse prognosis and cardiac damage. The enhanced inflammatory response in patients with sepsis promotes high expression of PTEN and impairs cardiac function. In the context of sepsis, blocking PTEN signalling can effectively lower the rate of T-cell death, increase T-cell proliferation, improve monocyte function [22][23][24][25], reduce the apoptosis of myocardial cells, and alleviate myocardial injury. In addition, this study revealed that independent risk variables, including the cTnl level, SOFA score, and APACHE II score, also influence the prognosis of patients with SIMI. The severity of sepsis increases with rising APACHE II and SOFA scores, which are less conducive to patient prognosis and recovery [26][27][28]. Elevated cTnI is strongly associated with the worsening of myocardial injury in sepsis patients and is a significant indicator of myocardial injury. Clinically, disease progression in SIMI patients can be detected through the APACHE II and SOFA scoring systems [29]. Moreover, the degree of myocardial injury can be assessed by detecting changes in cTnI levels, which more accurately determine the patient's condition and facilitate further treatment and improved prognosis [30][31][32].

In conclusion, high levels of blood PDK4, NLRP3, and PTEN in SIMI patients are associated with myocardial damage and are risk factors that influence the prognosis of SIMI patients. The prognosis can be improved by detecting changes in serum PDK4, NLRP3, PTEN and cTnI levels. However, this study still has limitations in terms of its experimental design. The sample size is relatively small, and the method is single. Changes in the serum factors of patients after improvement were not detected further, which may have led to bias in the sample size calculation results. Subsequently, multicenter validation will be adopted to study the working mechanism of serum factors in SIMI in detail and to complete the experimental steps, ensuring the scientific rigour of the research.

## Dodatak

### Conflict of interest statement

All the authors declare that they have no conflict of interest in this work.
